# Neandertal Cold Adaptation: Technological, Anatomical, and Physiological Responses to Cold Stress in One of Our Closest Fossil Relatives

**DOI:** 10.1002/ajhb.70150

**Published:** 2025-09-30

**Authors:** Trenton W. Holliday, Cara Ocobock, Libby W. Cowgill, Scott D. Maddux

**Affiliations:** ^1^ Department of Anthropology Tulane University New Orleans Louisiana USA; ^2^ Centre for the Exploration of the Deep Human Journey, University of the Witwatersrand Johannesburg South Africa; ^3^ Department of Anthropology and Eck Institute for Global Health University of Notre Dame South Bend Indiana USA; ^4^ Department of Anthropology University of Missouri Columbia Missouri USA; ^5^ Center for Anatomical Sciences, University of North Texas Health Science Center Fort Worth Texas USA

**Keywords:** BMR, body form, brown adipose tissue, climate, Pleistocene, TEE

## Abstract

Neandertals occupied western Eurasia for over 100 000 years, repeatedly enduring climates that ranged from seasonally cold to glacial. This paper reexamines the question of Neandertal cold adaptation using updated fossil, physiological, and archaeological evidence. While some populations lived outside glacial extremes, all faced periodic cold stress, and their survival depended on a diverse set of strategies. Technological buffers, including fire use, hide processing tools, and possible clothing and footwear, likely played a primary role in reducing cold exposure. Anatomically, Neandertals exhibit high body mass, broad trunks, and abbreviated limbs, consistent with thermoregulatory principles. The Neandertal nasal region, long considered paradoxical, now appears well suited to cold‐dry air‐conditioning; computational fluid dynamics and new endoscopic data support a functionally integrated nasal cavity with substantial internal surface area. Physiological adaptations remain inferential but plausible, including elevated basal metabolism, energy‐dense diets, and potential use of brown adipose tissue. These factors likely contributed to high total energy expenditures, enabling thermoregulation in demanding environments. Rather than a single trait or “signature” adaptation, Neandertals present an integrated response to cold stress shaped by geography, development, culture, and genetics. This holistic view reframes Neandertal biology as deeply thermally engaged and sets the stage for targeted tests of function and mechanism in future research.

## 1 Introduction

*Homo sapiens* thrives on every continent across a plethora of environmental conditions. However, our taxonomic order, Primates, is an almost exclusively tropical taxon (there are a few exceptions such as golden snub‐nosed monkeys [ *Rhinopithecus roxellana* ] and Japanese and Barbary macaques [ *Macaca fuscata* and *M. sylvanus* , respectively]). However, our hominin lineage, particularly Neandertals, did not remain in the tropics. The Neandertals are fossil hominins from western Eurasia who most likely split from our species during the Middle Pleistocene, around 500 000–700 000 years ago (Green et al. 2006; Noonan et al. 2006; Stringer 2012), or perhaps even earlier than 800 000 years ago (Gómez‐Robles 2019). While most researchers consider Neandertals to be a distinct human species (*H. neanderthalensis*), genetic evidence suggests that Neandertals successfully interbred with *H. sapiens* as well as with an enigmatic species known as the “Denisovans,” *H. longi*, or *H. juluensis*, who themselves also interbred with modern humans (Slon et al. 2018; Jacobs et al. 2019; Bae 2024; Bae and Wu 2024). Consequently, while Neandertals are extinct, their genetic legacy persists in people today.

**FIGURE 1:**
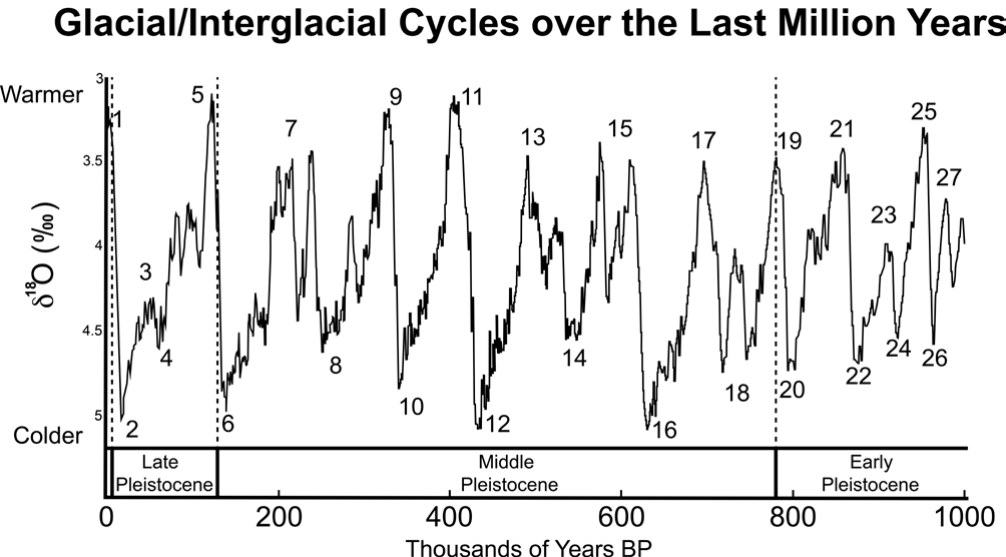
Global stacked benthic oxygen isotope (δ^18^O, a proxy for temperature) curve for the last million years. Marine isotope stages (MIS) are indicated by the numbers adjacent to the curve. Glacial phases are typically denoted by even numbers, while odd numbers tend to be interglacial phases. The coldest glacial phases over the last million years were MIS 16, 12, 6, and 2. Redrawn and modified from Lisiecki and Raymo (2005).

Neandertals' geographic range extended from the Iberian Peninsula in the west to the eastern shore of the Mediterranean, across the Zagros Mountains and into Central Asia at least as far as southern Siberia. To date, there is no evidence of Neandertals in Africa or South or East Asia. During the Middle and Late Pleistocene, Neandertals or their ancestors experienced at least eight glaciation events punctuated by shorter, warmer, interglacial phases (Figure 1). Temperatures during glacial maxima tended to be much colder than those we experience today. Tierney et al. (2020) estimate that during the Last Glacial Maximum, for example, average global temperatures were around 8°C (46° F)–much chillier than the twentieth century's average global temperature of about 14°C (57° F). Human populations during these time periods may have been marked by a distinct “ebb and flow” pattern of geographic occupation, moving in and out of more northern regions during the most severe glacial periods, which would have been characterized by advancing ice sheets (Hublin and Roebroeks 2009).

Given these generally colder climatic conditions, one would expect Eurasian Neandertals to have experienced long periods of intense cold that posed a challenge to their ancestrally tropically‐adapted anatomy and physiology. It is important to note that not all Neandertals lived during glacial periods, and many inhabited regions such as the southern Levant, which never experienced the steep reduction in temperature so characteristic of the higher latitudes of more continental Eurasia (CLIMAP 1976, 1981; Mellars 1996; Slimak 2024). However, even during interglacial phases, Neandertals across Eurasia would have faced seasonal cold, much like contemporary Eurasians—and their biology must have felt its influence. In fact, for many decades now paleoanthropologists have documented anatomical evidence for cold adaptation in Neandertals (Trinkaus 1981; Holliday 1997; Churchill 1998), while other researchers have made inferences about any underlying physiological adaptations they may have had to cold (Ocobock et al. 2021).

In this journal in 2002, Steegmann, Cerny, and Holliday reviewed the then current state of knowledge of Neandertal biological adaptation(s) to cold, approaching the question from multiple perspectives, including studies of cold acclimatization and/or biological adaptation in nonhuman primates, ethnographic/ethnohistoric evidence, and physiological studies of cold adaptation in recent humans and craniofacial and postcranial morphology of modern humans and Neandertals. Here we update their 2002 contribution with skeletal data from fossil and extant humans and new physiological data on recent humans. In this, we will cover four key ways modern humans (and likely Neandertals) adapt to cold: (1) technological or cultural means, (2) acclimatizations, or short‐term reversible biological responses to cold, (3) developmental adaptations, or adaptations that arise due to the impact of environmental factors during growth and development, and (4) biological adaptations, or genetic changes brought about via climatic selection across multiple generations.

## 2 Neandertal Cultural/Technological Adaptations to Cold

The primary means through which people today manage cold are cultural or technological, and this was likely the case with Neandertals, as well. Foremost among these adaptations is the controlled use of fire, which is likely the oldest and most effective innovation developed by humanity for providing heat. Some evidence, albeit controversial, suggests that controlled use of fire may date back as early as one million years ago in Africa (Gowlett et al. 1981; Brain and Sillent 1988; Berna et al. 2012). It is therefore unsurprising that Neandertals retained their ancestors' technology; hearths with concentrations of charcoal are common features in Neandertal sites (James et al. 1989; Henry 2017; Wragg Sykes 2020). While Neandertal hearths are typically simple constructions consisting of fuel laid out and burned on a flat surface, archaeologists working in unusually well‐preserved, presumably Neandertal Mousterian contexts at the site of Abric Romaní in Catalonia have uncovered examples of much more complex imbedded hearths with subterranean channels dug to feed oxygen to the fire (Courty et al. 2012; Vallverdú et al. 2012). While controversial, some have argued that Neandertal production of birch tar for the hafting of stone points implies extremely sophisticated knowledge of fire and its properties, given that the birch tar adhesive requires anaerobic conditions for its manufacture (Grünberg 2002; Kozowyk et al. 2017; Schmidt et al. 2019).

**FIGURE 2:**
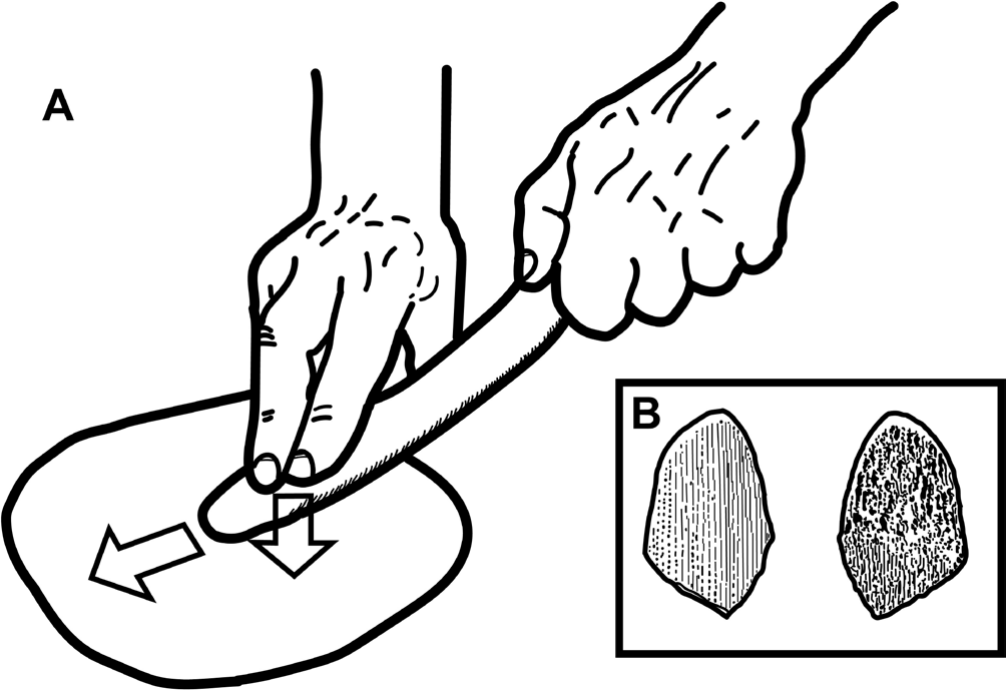
(A) Reconstruction of how a *lissoir*, a Neandertal artifact made from the rib of a large ungulate and likely a leatherworking tool, was used to burnish leather. The downward pressure accompanied by forward motion frequently led to breaking the tip of the tool off. Redrawn from an illustration by the Abri Peyrony‐Pech‐de‐l'Azé I Projects. (B) Drawings of the superficial (L) and deep (R) surfaces of the broken‐off tip of a bone *lissoir* (AP‐4493) from Middle Paleolithic levels at Abri Peyrony. Redrawn from Soressi et al. (2013).

The use of clothing as protection against the cold is another important consideration. Genetic data suggest that our ancestors lost most of their long, fur‐like hairs by at least 1.2 million years ago (Rogers et al. 2004), and fossil evidence indicates it may have been earlier still (Franciscus and Trinkaus 1988). Like modern humans, Neandertals were mostly hairless, with most of their bodies covered only by fine vellus hairs. As such, they would almost certainly have needed to wear clothing to survive in the colder climes of Eurasia. Clothing would have been made primarily, if not exclusively, from perishable materials, making it difficult to pinpoint its date of origin. However, tools associated with clothing manufacture are often made from more durable materials, providing indirect evidence. In this light, straight‐edged stone tools known as scrapers, long thought to have been used to scrape hides, have been recovered in archaeological association with Neandertal remains since their recovery at La Naulette, Belgium, in 1866 (Dupont 1866); they remain the most common lithic artifact found in Neandertal sites. One challenge, however, is that these so‐called scrapers were likely multifunctional tools, useful for a variety of tasks beyond clothing manufacture, or were a source for small flakes. A more tantalizing bit of evidence for Neandertal clothing manufacture is the recovery of *lissoirs*, or burnishers, an artifact often made from cervid or bovine ribs frequently recovered from Neandertal contexts (Soressi et al. 2013; Wragg Sykes 2020). In fact, these tools may have been invented by Neandertals (Soressi et al. 2013). These artifacts are directly comparable to tools used by modern leatherworkers to rub hides to make them more waterproof and/or polished (Figure 2A). Recovered *lissoirs* show signs of extensive use and often show damage consistent with breakage from manual pressure (Soressi et al. 2013), (Figure 2B).

Eyed needles are considered the best evidence for clothing manufacture, as they are used to make fitted garments. While no eyed needles have been found in European Neandertal contexts, bone ones were recovered from Denisova Cave at ca. 50 000 years ago (Shunkov et al. 2020), a site periodically inhabited by Neandertals at that time. Their discovery raises the possibility that at least some Neandertals, particularly those in cold environs like southern Siberia, had developed or adopted this technological innovation, producing tailored clothing to deal with the extreme environmental cold they experienced.

Evidence for Neandertal footwear remains inconclusive. Footprints attributed to Neandertals have largely been recovered from within caves, although two recent discoveries were made in coastal or beach contexts (Duveau et al. 2019; Mayoral et al. 2021). However, all Neandertal footprints thus far discovered were made exclusively by barefoot individuals. We must be mindful that it is possible shoes were removed inside caves due to their stable internal temperatures. Might Neandertals have donned some form of footwear when negotiating cold external environments of Pleistocene Eurasia? Trinkaus (2005) examined the lesser toes' pedal phalanges of traditionally shod and unshod humans. People who wear stiff‐soled shoes have less muscular foot phalanges than those who remain barefoot or who wear soft, flexible footwear (e.g., moccasins), who tend to have more muscular lesser toes. In late Paleolithic contexts, Trinkaus found that Neandertals had more muscular lesser toe bones than early modern Europeans, leading him to hypothesize that Neandertals did not wear stiff‐soled shoes. He could not, however, rule out the possibility that they wore moccasin‐like shoes, at least outside of cave environments.

Another dataset used to investigate the origins of clothing involves genetic estimates of when human head lice ( *Pediculus humanus capitis* ) and body lice (*Pediculus humanus corporis*) diverged. Since body lice lay their eggs exclusively in clothing, clothing must antedate this split. Genetic estimates place this divergence between approximately 83 000 and 170 000 years ago (Toups et al. 2011). This could suggest that the apparent waves of *H. sapiens* out of Africa we see at around 100 000 and 60 000 years ago (Mellars 2006; Henn et al. 2012) were of people who were already wearing clothes. That said, it is also possible that body lice evolved among Neandertals and/or Denisovans before being transmitted to *H. sapiens* populations expanding into Eurasia. In the end, the bulk of data suggest that Neandertals wore clothing, albeit more loose‐fitted clothing, for most of their existence in Eurasia. However, in particularly cold environments such as southern Siberia, Neandertals may have developed more tailored garments as an adaptive response to extreme temperatures.

## 3 Neandertal Biological Adaptations to Cold: Body Size and Proportions

**FIGURE 3:**
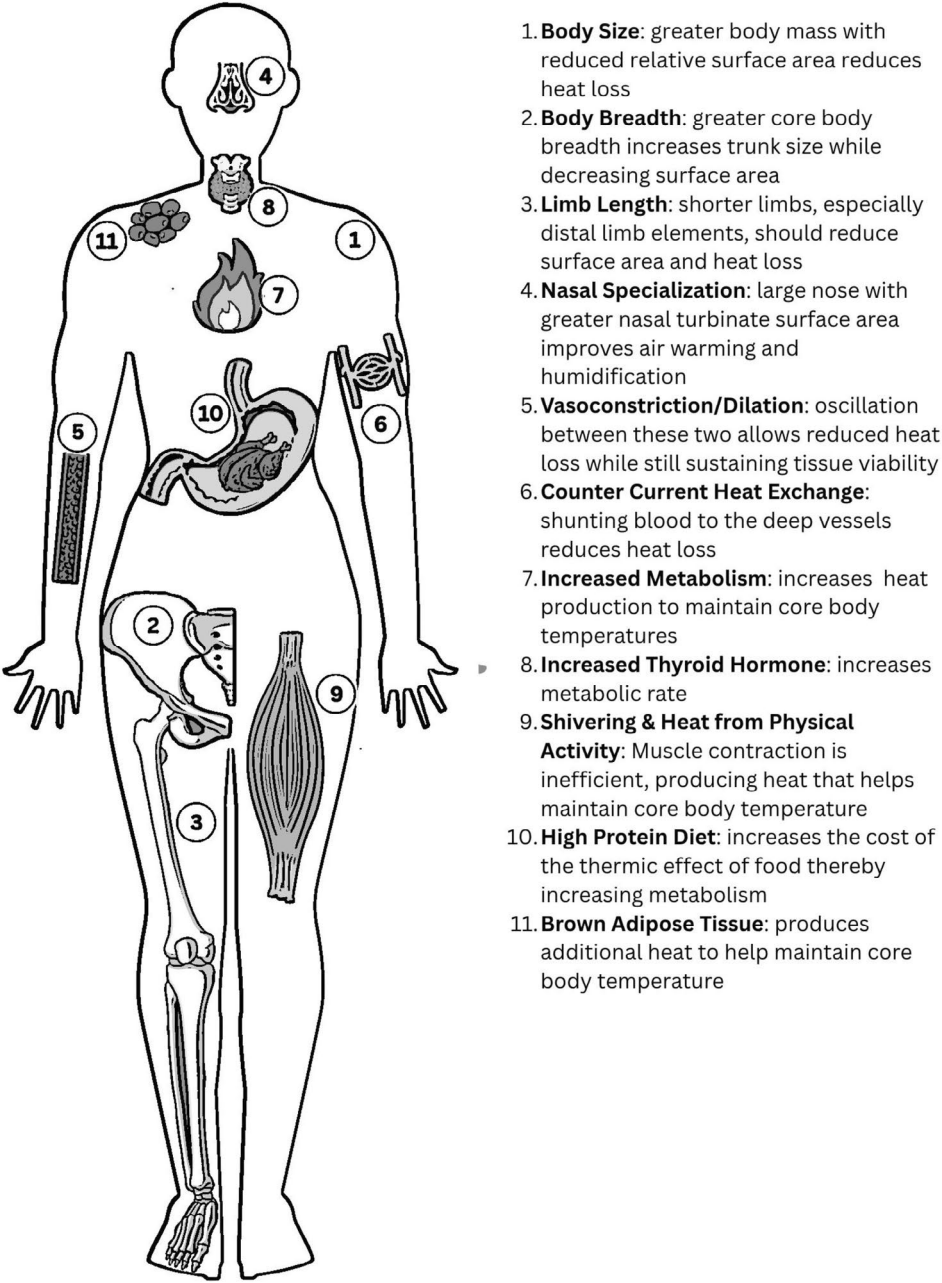
A schematic diagram of the human body showing the potentially cold‐adapted aspects of Neandertal biology investigated in this contribution.

As shown in Figure 3, Neandertals exhibit numerous potential anatomical adaptations to cold environments, including larger body size, abbreviated limb proportions, and distinctive anatomy of their facial skeleton. In terms of body size and limb proportions, many widespread endothermic species follow predictable patterns across their species' geographic range—a phenomenon known as ecogeographical patterning. These patterns were first formalized in the nineteenth century as ecological rules, specifically Bergmann's (1847) and Allen's (1877) Rules. Bergmann's Rule states that within a widespread endothermic species, those individuals and populations in colder regions tend to have elevated body mass compared to those individuals and populations in warmer regions. Allen's Rule describes a pattern where individuals and populations in warmer environments display longer extremities, while their conspecifics in colder areas exhibit shorter extremities.

The conventional explanation for these patterns is based on the relationship between surface area and volume ratios (SA:V) and their impact on temperature regulation in endothermic animals. For example, regarding Bergmann's Rule, when two objects share the same shape but differ in size—for example, a tennis ball and a basketball—the larger object will have a smaller SA:V than the smaller one. According to Fourier's Law of heat flow, heat loss in animals is directly proportional to their surface area, meaning that a bigger animal, with its lower relative surface area, should retain heat better than a smaller animal with a higher SA:V. Heat retention would be advantageous in cold climates, while the shedding of excessive body heat would be adaptive in hot environs. This provides an advantage in cold climates, whereas smaller animals with higher SA:V ratios in hot environments can more efficiently dissipate excess heat. Following this logic, within a widespread species of endothermic animals, we expect those groups and individuals in colder regions to be larger than those individuals or populations in hotter regions.

In this light, Allen's Rule further refines this concept by explaining deviations from Bergmann's Rule. Specifically, if two animals are the same size, but one has longer extremities than the other, then the animal with longer limbs will have a higher SA:V ratio. This is due to the fact that limbs, with their much narrower cross‐sections, show higher relative surface areas than the trunk. Consequently, within a widespread endothermic species, those individuals and populations in hotter regions are expected to evince longer extremities than those in colder regions.

While this theoretical explanation has been called into question numerous times (Rosenzweig 1968; McNab 1971; Geist 1987; Ashton 2002; Olson et al. 2009; Nunes et al. 2017; Bogin et al. 2022; and see below), a key point to recognize is that these ecological rules are merely empirical observations of nature, and as such are not reliant on any specific theoretical explanation for their validity. In fact, multiple studies have shown that a variety of endothermic species, both avian and mammalian, show adherence to Bergmann's and/or Allen's Rule (Cowan 1936; Young and Goldman 1946; Hamilton 1961; Murphy 1985; Aldrich and James 1991; Graves 1991; Fooden and Albrecht 1993; Ravosa 1998; Clarke and O'Neil 1999; Ashton et al. 2000; Yom‐Tov 2001; Meiri and Dayan 2003; Blackburn and Hawkins 2004). As first pointed out by Coon (1962) and Badoux (1965), Neandertals, and especially European Neandertals, are no exception to this rule, displaying a host of anatomical features consistent with these ecological rules. For example, Neandertals tend to have considerably larger femoral heads than living people. While some of this can be attributed to a bony response to higher activity levels during growth and development than seen today, as a weight‐bearing joint, femoral head size is arguably the best skeletal measurement for estimating hominin body mass in hominins (McHenry 1992; Jungers et al. 2016; Ruff et al. 2018), and therefore is reflective of Neandertals' elevated body mass. In fact, while on average shorter than modern humans, body mass estimates indicate that Neandertals were nonetheless heavier. Ruff et al. (1997) found that recent humans from high latitudes (*n* = 53) have a mean body mass of 58.6 kg, while the European Neandertals (*n* = 18) had an average body mass of 73.6 kg, approximately 25% larger. More recent estimates are consistent with this figure—Plavcan et al. (2014) calculate European Neandertal body mass (*n* = 16) at ca. 72.0 kg, which is slightly below Ruff's estimate but still about 20% larger than that of recent high latitude humans. Bergmann's Rule would predict that West Asian Neandertals would be smaller than their European conspecifics, and this is the case. Ruff et al. (1997) calculate a mean mass of 71.6 kg for West Asian Neandertals (*n* = 9), while Plavcan et al. (2014) provide a somewhat lower estimate at 70.2 kg—about 2.5% smaller than their European counterparts.

Another Neandertal feature potentially related to cold adaptation is their broad trunks. Albeit with limited samples, across their geographic range, Neandertals appear to have been characterized by large bi‐iliac (pelvic) breadths, reflecting their larger body size (Holliday 2012). One issue with attributing Neandertals' broad trunks to cold adaptation, however, is that wide pelves appear to be the ancestral condition for the genus *Homo*. Middle Pleistocene specimens from Jinnuishan, China, and Atapuerca, Spain, show markedly wide pelves (Rosenberg et al. 2006; Holliday 2012), as does BSN49/P27, an early Pleistocene presumed *Homo* pelvis from Gona, Ethiopia—a decidedly tropical location (Simpson et al. 2008; C. Ruff 2010). The fossil record seems to hint that it is *H. sapiens* , with our narrow pelves, who represent the derived condition within our genus.

Neandertals have also long been hypothesized to possess voluminous ribcages, a feature that likely positively covaries with pelvic breadth (Churchill 1998; Franciscus and Churchill 2002; López‐Rey et al. 2025; and see below). Specifically, the second rib, one of the few ribs that can be reliably identified without the need for seriation, exhibits more anteroposterior expansion compared to living humans (Churchill 1994a, 1994b). Unfortunately, due to the relative fragility of ribs, only two nearly complete Neandertal ribcages have thus far been recovered: Kebara 2 from Israel and Shanidar 3 from Iraq. López‐Rey et al. (2025) have recently compared the virtually reconstructed ribcage of Shanidar 3 to the previously reconstructed ribcage of Kebara 2 (Gómez‐Olivencia et al. 2018), analyzing them via 3D morphometrics. They found that both Kebara 2 and Shanidar 3 had “bell‐shaped” thoraces long said to characterize Neanderthals, as opposed to the more “barrel‐shaped” thoraces seen in *H. sapiens* . What does “bell‐shaped” mean? It means that like a bell, the ribcages of Neandertals are relatively broad in their superior aspect (unlike the cone‐shaped ribcages of the African apes) yet continue to increase in breadth as one moves down the thoracic column toward the lumbar region and pelvis. In contrast, the more barrel‐shaped ribcages of *H. sapiens* taper in breadth as one moves from the middle rib cage (ribs 5–7) toward the pelvic girdle.

In terms of ribcage shape, López‐Rey et al. (2025) note that both Shanidar 3 and Kebara 2 cluster separately from most *H. sapiens* , although they lie morphologically closer to cold‐adapted *H. sapiens* individuals. Given that both these specimens originate from Western Asia, it is possible that Neandertal ribcage morphology does not primarily reflect cold adaptation but is instead associated with larger lungs and increased vital capacity, potentially to support their metabolically expensive bodies (Churchill 2006, 2014). However, it would not be surprising if the rib cages of European Neandertals were even more extreme in terms of size and/or bell‐like shape; future discoveries of relatively complete European Neandertal skeletons could provide a test of this hypothesis. Multiple muscles of the body core, as well as both the upper and lower limbs, span the region between the pelvis and ribcage. It is therefore likely that the inferiorly broad, bell‐like shape of the Neandertal thorax is functionally integrated with their wide pelves. In contrast, modern human ribcages taper in breadth inferiorly (i.e., are barrel‐shaped) to integrate more effectively with our species' narrow pelves.

Trinkaus (1981) showed that Neandertals were characterized by high claviculo‐humeral indices (clavicle length/humerus length), which at the time were thought to reflect climatic adaptation. However, this is unlikely, for three reasons. First, in people today the index is not correlated with climatic variables (Trinkaus 1981). Second, Trinkaus et al. (2014) showed that differences in this index between Neandertals and modern humans are due to Neandertals' shorter humeri, not elongated clavicles, and third, clavicle length is more correlated with overall muscularity than with body breadth (Roberts 1978; Crognier 1981; Holliday 1995).

The femoral head is often used to estimate body mass, and the relationship between femoral head diameter and femoral length has therefore been used as a measure of relative body linearity (Ruff 1987, 1990, 1994; Franciscus 1989; Jungers 1991; Holliday 1997; Holliday and Hilton 2010; Holliday 2012). A key advantage of this metric is it requires just one bone for investigation. Since Bergmann's Rule predicts body mass increases with cold, and Allen's Rule suggests extremities become shorter over the same geographical gradient, a skeletal measure of body size (femoral head), scaled to a reflection of body linearity (femoral length), should exhibit ecogeographical patterning. In fact, Holliday and Hilton (2010) found that three circumpolar Inuit (Ipiutak, Tigara, and Koniag) samples from Alaska had higher femoral head‐to‐femoral length indices than European, North African, and sub‐Saharan African samples, following ecological predictions. When a small sample of Neandertals (La Chapelle‐aux‐Saints 1, La Ferrassie 1, Neandertal 1, Shanidar 4 and 5, Spy 2, and Tabun C1; Holliday 1995) is compared to the Inuit samples, four of the six Neandertal individuals' indices fall above the Inuit median values—some substantially so; only Tabun C1 and Shanidar 5, both West Asian specimens, fall below the Inuit medians. Thus, Neandertals are characterized by femora with bigger heads relative to their length than is typical among recent modern humans, even those from circumpolar groups.

Another skeletal manifestation of Allen's Rule is the relative length of the limbs compared to trunk height, with shorter limbs expected in cold‐adapted groups. Consistent with this expectation, Neandertal limb‐to‐trunk height ratios fall below the *H. sapiens* average, indicating relatively abbreviated limb lengths (Holliday 1995, 1997). In addition, intralimb proportions, or differences in limb segment lengths within each limb, are also correlated with climatic variables in recent humans (Trinkaus 1981). Neandertals evince low crural (tibia length/femur length) and brachial (radius length/humerus length) ratios, indicating that the distal limb segments are foreshortened relative to the proximal ones—a trait long associated with modern humans inhabiting cold climates (Bello y Rodriguez 1909). Note, too, that Neandertals show regional variability in intralimb indices. Neandertals in both Europe and West Asia show small crural indices, but West Asian Neandertals have higher brachial indices (Franciscus 1989), likely reflecting climatic differences between these regions. An intriguing question remains: would Neandertal skeletons from southern Siberia, if more complete specimens were available, display even more cold‐adapted features than those found in Europe and the Levant?

**FIGURE 4:**
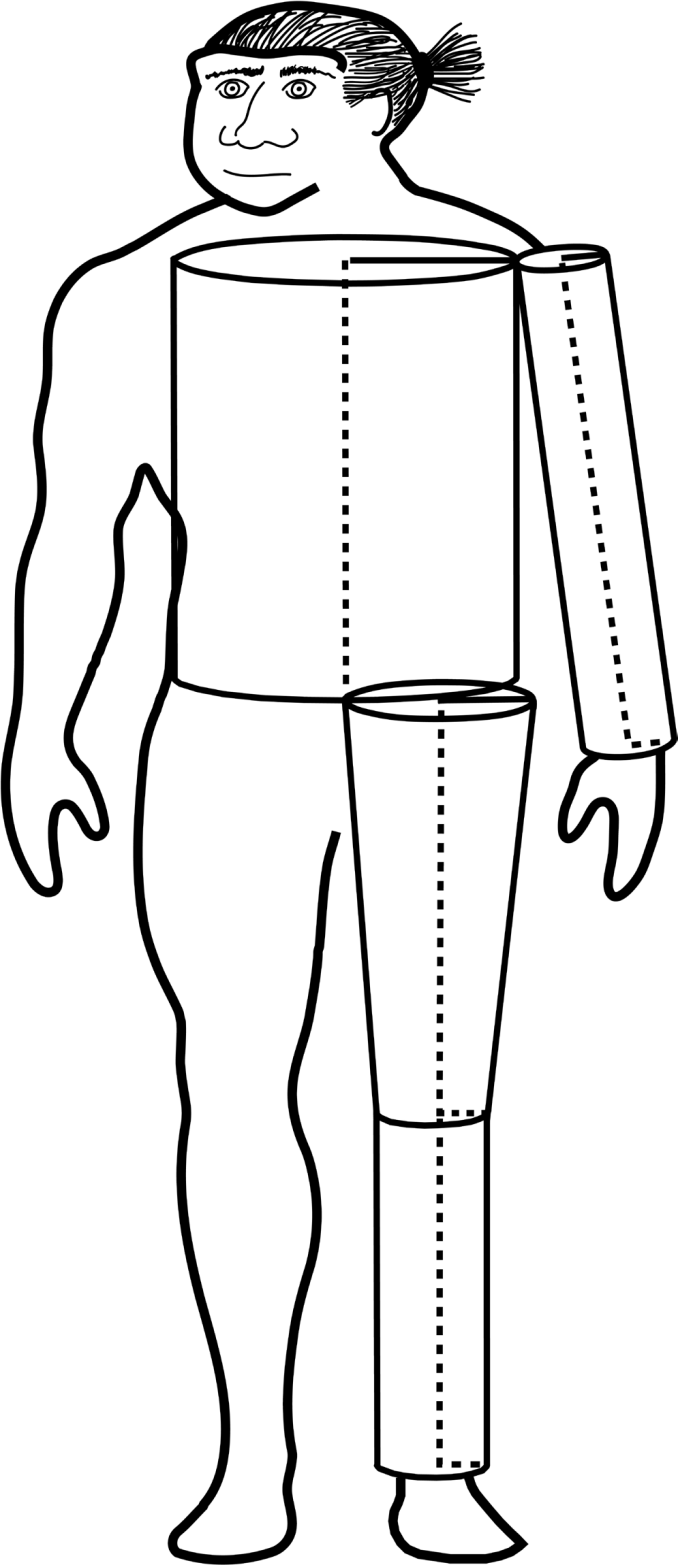
A schematic drawing of a Neandertal, showing how body volume and surface area were calculated following Kasabova and Holliday (2015). The trunk is modeled as a cylinder with bi‐iliac breadth as its diameter and a height equal to acromial height minus lower limb length. The upper limb is modeled as a cylinder, with humeral biepicondylar breadth as its diameter and a height equal to the summed lengths of the humerus and radius. The thigh is modeled as an inverted frustrum with a height of femoral length, a proximal diameter equal to bitrochanteric breadth divided by two, and a distal diameter equivalent to femoral biepicondylar breadth. The distal lower limb is modeled as a cylinder with a height equivalent to tibial length and a diameter equal to tibial plateau breadth. Redrawn and modified from Kasabova and Holliday (2015).

All the aforementioned skeletal features align with the expectation that Neandertals, particularly European Neandertals, evolved a more cold‐adapted body size and shape in response to the colder climes of Pleistocene Eurasia. Two issues, however, temper this argument. First, intralimb proportions do not appear to have much of an effect on the body's surface area‐to‐volume (SA:V) ratio. In 2015, Kasabova and Holliday modeled the surface area and volume of the human body using a combination of skeletal and anthropometric data. Specifically, their model used a set of cylinders and frustra to calculate volume and surface areas for the trunk and limb segments. These shapes, when arranged in anatomical position, resembled the “Tin Man” from “The Wizard of Oz,” leading Kasabova and Holliday to refer to their method as the “Tin Man” model (Figure 4). The “Tin Man” method yielded body surface area and volume estimates comparable to those of more rigorous anatomical techniques. When scatter plots of body volume on body surface area were generated, there was separation with overlap between high latitude (cold‐adapted) versus low latitude (heat‐adapted) recent humans. The least‐squares slopes of these two groups were not significantly different, but their intercepts were, with the cold‐adapted group's being higher. Thus, heat‐adapted recent humans showed greater body surface areas relative to body volume, while higher‐latitude, cold‐adapted individuals showed lower body surface areas relative to body volume (Kasabova and Holliday 2015). That said, this separation was primarily due to pelvic breadth differences; changes in intralimb proportions had a negligible effect on the body's relative surface area.

In 2019, Holliday and Kasabova applied their method to estimate body surface area and volume of several late Pleistocene fossils from Eurasia and northern Africa, including three Neandertals (Holliday and Kasabova 2019). Fossils were compared to high latitude/cold‐adapted (*n* = 133) and low latitude/heat‐adapted (*n* = 192) recent human skeletons. The Neandertals fell closer to the high latitude recent human body volume‐to‐surface area regression line and farther from the heat‐adapted recent human line; they have smaller body surface areas relative to their body volumes. As before, much of the variation in Neandertal relative body surface area is attributable to their broad pelves, consistent with the earlier and widely cited work of Christopher Ruff (1991, 1994). Ruff modeled the human body as a cylinder with bi‐iliac breadth as its diameter and stature as its height, originally as a non‐biomechanical method for estimating fossil hominin body mass. Analyzing relative surface area derived from his method, he argued that differences in pelvic breadth have a much greater impact on relative body surface area than do changes in limb length or body height. The more anatomically accurate “Tin Man” method yields the same result.

The second factor complicating the argument that Neandertal body proportions resulted from selection for cold adaptation is the potential influence of ambient temperature on limb length during critical periods of growth and development. Importantly, growth experiments conducted on mice by anatomist Maria Serrat were able to show exactly how limb foreshortening arises in young animals exposed to cold (Serrat et al. 2008; Serrat 2013). Specifically, distal limb foreshortening results from a conserved pan‐mammalian physiological response in which blood vessels in the limbs vasoconstrict when exposed to cold. This facilitates survival by maintaining a steady blood supply to the vital organs of the trunk, head, and neck, while simultaneously reducing circulation to the extremities, where blood would otherwise cool more rapidly. Serrat demonstrated that if this vasoconstriction occurs frequently during key developmental stages, the reduced blood flow to the extremities inhibits limb growth at the epiphyseal plates, leading to permanently shortened limb segments. Thus, rather than being the result of selection, the shorter distal extremities of Neandertals could simply reflect prolonged exposure to cold during their growth and development. However, this explanation alone does not fully account for the Neandertal pattern. In modern humans, trunk width in immature individuals displays a strong correlation with latitude during the first year of life (Cowgill et al. 2012). Furthermore, Neandertal fetal skeletons show clear distal foreshortening, suggestive of either a strong genetic component (Weaver et al. 2016) or alternatively reflecting in utero effects of maternal cold and/or hypovitaminosis D stress (McGrath et al. 2005).

## 4 Neandertal Biological Adaptations to Cold: Neandertal Facial Morphology

**FIGURE 5:**
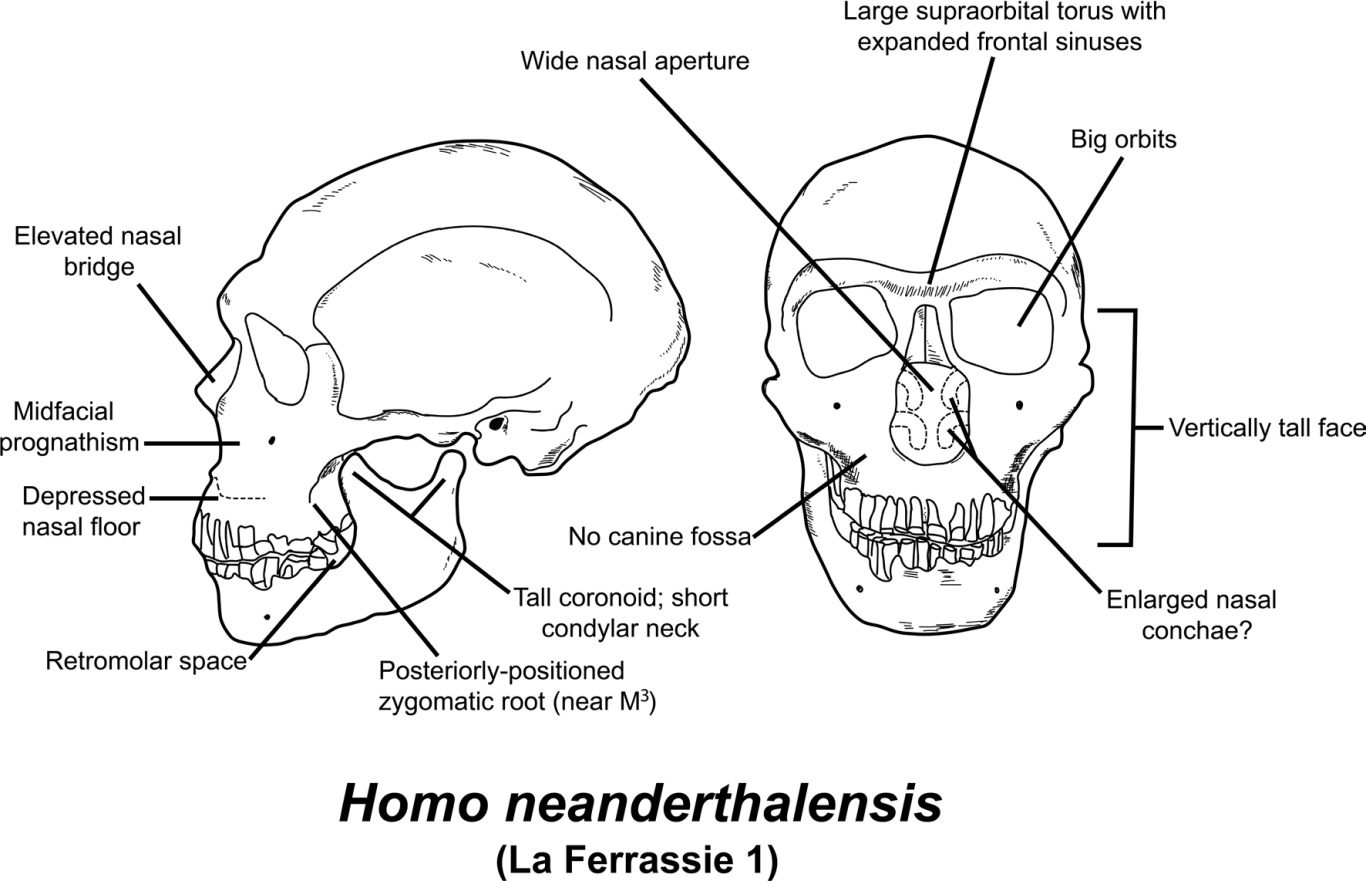
Left lateral (L) and anterior (R) views of the La Ferrassie 1 Neandertal skull, showing craniofacial features discussed in the text. Modified from Holliday (2023).

As evident in Figure 5, the form of the Neandertal facial skeleton is distinctive from that of both its presumed ancestors (e.g., *H. heidelbergensis*) and *H. sapiens* (Trinkaus 2006). Specifically, Neandertals are characterized by large orbits topped by prominent supraorbital tori (brow ridges), which are more pronounced than those of recent modern humans, but smaller than those of their presumed ancestors. As in *H. sapiens* , Neandertal brow ridges project near the midline and taper laterally (Athreya 2009, 2012). Neandertal faces are vertically “tall” and project anteriorly near the midsagittal plane, a feature known as midfacial prognathism. In contrast to *H. heidelbergensis*, Neandertals possess zygomatic bones (cheekbones) that are positioned more posteriorly, resembling the condition seen in *H. sapiens* (Trinkaus 1987; Maureille and Houët 1997). Their large anterior teeth are rooted in a broad anterior palate, above which is a wide nasal aperture, which lies in front of a frequently bi‐level, or depressed, nasal floor (Franciscus 2003; Holton and Franciscus 2008). Neandertal maxillae lack canine fossae and house large sinuses, as do the ethmoid, sphenoid, and frontal bones (Zollikofer et al. 2008; Buck et al. 2019). The distal ends of the nasal bones, when preserved, project nearly horizontally, and the rami of their long, chinless mandibles have high coronoid processes and relatively short condylar necks (Trinkaus 1983; Rak et al. 2002). The Neandertal posterior dentition is reduced relative to that of their presumed ancestors; this, coupled with a narrower mandibular ramus, leads to the presence of a space (retromolar space) between the distal margin of their mandibular third molars and the anterior margin of the ramus (Franciscus and Trinkaus 1995).

While there is broad consensus that the Neandertal facial configuration is unique, the evolutionary mechanisms that produced it remain a topic of debate (Smith et al. 2005; Tattersall and Schwartz 2006). Researchers differ in their interpretations of the relative contributions of adaptive and stochastic (random) forces. For example, Weaver et al. (2007) analyzed 37 standard craniometric measurements, encompassing both facial and neurocranial features, from W. W. Howells' global dataset of a large (*n* = 2524) sample of modern humans and 20 Neandertals. With a battery of statistical tests drawn from quantitative‐ and population‐genetic theory, they argued that purely random genetic changes (drift) could account for craniofacial differences between Neandertals and modern humans.

However, cold adaptation has been advanced as a possible explanation for some Neandertal craniofacial features. In the human cranium, for example, the paranasal sinuses (air‐filled cavities or cells within the frontal, ethmoid, sphenoid, and maxillary bones) are produced via differential growth and communicate with the nasal cavity via small openings. Many different functional and non‐functional explanations for the sinuses have been put forward (Márquez 2008; Butaric and Maddux 2016; Kim et al. 2022), including assertions that the sinuses act to insulate the brain from environmental insult, such as extreme temperatures. For example, in his 1944 description of the Saccopastore Neandertal, Sergio Sergi called attention to the specimen's large paranasal sinuses, suggesting their size was attributable to cold adaptation (Sergi 1944). Similarly, Coon (1962) agreed, arguing that large sinuses belonged to a suite of Neandertal characters “that gave them an advantage for survival in the cold” (Coon 1962: 522). However, more recent studies have shown that Neandertal sinuses are not particularly voluminous or “hyperpneumatized”, once adjusted for overall cranial size (Zollikofer et al. 2008; Rae et al. 2011; Buck et al. 2019). Further, it has been noted that sinus size generally does not exhibit strong climatic signals among extant *H. sapiens* , with populations from temperate regions typically possessing larger sinuses than those from both circumpolar and equatorial regions (Koertvelyessy 1972; Shea 1977; Hanson and Owsley 1980; Butaric et al. 2010; Rae et al. 2011; Holton et al. 2011; Butaric 2013; Noback et al. 2016), undermining the use of sinus size to infer climatic adaptation in Neandertals (Holton et al. 2011; Noback et al. 2016).

Like the sinuses, the nose has also received considerable attention in relation to potential cold adaptation in Neandertal facial morphology (Franciscus 1995, 1999, 2003; Holton and Franciscus 2008; Márquez et al. 2014; Wroe et al. 2018). This is because, among recent modern humans, the nose and surrounding midface exhibit the strongest correlation coefficients with environmental variables such as temperature and humidity (Thomson and Buxton 1923; Davies 1932; Wolpoff 1968; Hiernaux and Froment 1976; Crognier 1981; Franciscus and Long 1991; Noback et al. 2011; Evteev et al. 2014; Maddux et al. 2017).

These strong statistical associations, in turn, almost certainly relate to the important role of the nose in mediating exchanges of heat and moisture between the body and the available supply of environmental air. Indeed, to maintain effective pulmonary gas (O_2_/CO_2_) exchange, inspired air must enter the lungs at core body temperature and fully saturated with water vapor. As ambient air conditions in most areas of the world are typically below 37°C and 100% relative humidity, the respiratory system is generally required to add heat and/or moisture to inspired air until it matches pulmonary conditions. Importantly, the nose acts as the body's primary “air‐conditioner”, accounting for approximately 90% of total respiratory heat/moisture exchanges (Naftali et al. 2005; Issakhov et al. 2021). As clinical studies have demonstrated that differences in nasal morphology directly influence heat and moisture exchange capabilities (Lindemann et al. 2016; Inthavong et al. 2019; Russel et al. 2024), it is widely accepted that ecogeographic variation in modern human nasal morphology reflects varying demands for respiratory heat/moisture exchange across different climatic regions (see Bastir et al. 2022 for a review).

Compared to both earlier archaic and recent humans, the Neandertal external nose is unique in that it is characterized by a combination of projecting nasal bones in conjunction with a nasal aperture that is both tall and wide (Franciscus 1995, 1999; Márquez et al. 2014). In recent modern humans, projecting nasal bones have been shown to occur primarily in populations indigenous to cold and/or dry environments, who also tend to possess relatively tall and *narrow* nasal apertures. In contrast, recent human populations from more tropical environments typically exhibit less projecting nasal bones along with relatively short and *wide* nasal apertures (Williams 1956; Carey and Steegmann 1981). Thus, Neandertals display a paradoxical combination of external nasal features, with their projecting and tall external noses appearing consistent with expectations for cold adaptation, but their wide nasal aperture breadths appearing more similar to extant populations from tropical environments (Holton and Franciscus 2008).

While it is widely recognized that heat and moisture transfer predominantly occurs within the internal nasal cavity (Cole 1982; Mlynski et al. 2001; Naftali et al. 2005), it has been suggested that a more projecting external nose may facilitate inspiratory air‐conditioning by causing the inflowing airstream to diffuse as it enters the nasal cavity, thus leading to greater contact with internal nasal mucosa and enhanced heat and moisture transfer (Mlynski et al. 2001; Zhu et al. 2011). Further, it has also been suggested that greater nasal projection may also facilitate mucosal recapture of heat and moisture from expired air, thus limiting the amount of heat and water lost to the environment during exhalation (Franciscus and Trinkaus 1988; Yokley 2009; Noback et al. 2011). This latter phenomenon can be demonstrated by exhaling onto a mirror; significantly less moisture condenses when exhaling through the nose compared to exhaling through an open mouth. Thus, given that all Neandertals would have invariably breathed cold‐dry air (cold air is less water‐saturated and therefore inherently dry; Lutgens et al. 2019) during winter months, the presence of a projecting external nose in Neandertals may reflect cold‐mediated pressures for both inspiratory air‐conditioning and expiratory heat/moisture retention.

Since the vast majority of respiratory heat and moisture exchange occurs within the mucosa‐lined nasal cavity, variation in internal nasal morphology has been shown to exhibit strong correlations with climatic variables across recent modern humans (Yokley 2009; Noback et al. 2011; Evteev et al. 2014; Maddux et al. 2017; Maréchal et al. 2023; Bastir et al. 2024). The internal nasal cavity is packed with bony structures that significantly increase its relative surface area (Yokley 2009; Marks et al. 2019). These structures include the nasal septum, which divides the nasal cavity into left and right halves, as well as the superior, middle, and inferior nasal conchae, also known as turbinates. All of these structures, along with the surrounding walls of the nasal cavity, are covered by highly vascularized nasal mucosa. As the arterial blood supply to this mucosa originates in the body core, the mucosa provides an ample source of heat when inspired air comes into contact with it. Additionally, the nasal mucosa is also capable of producing vast quantities of mucus, providing the source of moisture needed to humidify inspired air. What has flummoxed many researchers with regard to the Neandertal internal nose is that in recent humans who live in cold environments, the nasal cavity tends to be relatively narrow (Franciscus 1995; Yokley 2009; Noback et al. 2011; Maddux et al. 2017). This makes sense, as a narrower nasal cavity increases the amount of mucosal surface area relative to the total volume of the nasal airways (i.e., a higher SA:V ratio). This increased SA:V ratio thus forces more of the air in each breath to come into contact with the warm and wet mucosa, facilitating heat and moisture exchange (Yokley 2009; Maddux et al. 2016), a critical adaptation in environments characterized by cold (and therefore dry) air like Pleistocene Eurasia. This raises two key questions: (1) Why were Neandertal noses so wide, and (2) could their wide noses have still adequately conditioned cold‐dry air?

With regard to the first question, work by Holton and Franciscus (2008) suggests an answer. Here, it is important to note that, in addition to being wide, the Neandertal nasal cavity is also anteroposteriorly long, a feature tied to the retention of facial prognathism (projection) among Neandertals from their Middle Pleistocene ancestors (Coon 1962; Franciscus 1995; Trinkaus 2003). Using a large sample of Pleistocene fossil specimens in conjunction with data from a longitudinal study of modern human facial growth, Holton and Franciscus (2008) were able to show that nasal breadth dimensions are developmentally constrained by facial projection, such that growing a more prognathic face inherently results in a wider nose. As a consequence, they suggested that the characteristic midfacial prognathism of Neandertals likely precluded nasal narrowing as an adaptive response to cold‐induced respiratory pressures. Indeed, the relatively narrow noses of modern humans indigenous to cold‐dry environments may represent a novel adaptive strategy that only became possible following the evolution of a retrognathic facial skeleton, one of the defining features of our species (Lieberman et al. 2002; Trinkaus 2003; Stringer and Buck 2014).

While facial growth dynamics may have prevented Neandertals from evolving narrow noses, it is also possible that Neandertals actually needed wider noses. Given the nose's superior capacity for heat and moisture exchange, during normal unlabored breathing (eupnea), humans typically breathe entirely through their nose rather than through the mouth (Ingelstedt 1970). Thus, in addition to air‐conditioning, the nose must also facilitate the intake of enough air with each breath to meet fundamental metabolic requirements for oxygen consumption. This has led to the formulation of the “respiratory‐energetics hypothesis,” which postulates that nasal size is primarily driven by metabolic requirements for oxygen intake (Hall 2005; Bastir et al. 2011, 2020; Kelly et al. 2023). For example, in a pair of ontogenetic studies, Holton et al. (2014, 2016) found growth in nasal size predictably followed increases in body size in both males and females. Further, these studies found that males begin to develop significantly larger noses than females at the onset of puberty, coinciding with rapid increases in male body size and associated oxygen intake requirements during adolescence. Moreover, these studies found nasal growth tracked adolescent increases in male body size more closely than the surrounding face, suggesting that nasal morphology may be more inextricably constrained by metabolic demands than other aspects of the craniofacial skeleton. Thus, given that Neandertals were large‐bodied and would have undoubtedly had high metabolic requirements (see next section for details), their unusually tall and wide nasal morphologies may simply reflect a need for an exceedingly large nose capable of transmitting enough air in each breath to provide their bodies with sufficient oxygen (Froehle et al. 2013; Holton et al. 2014; Wroe et al. 2018).

Regarding the second question, would Neandertal noses have been able to adequately condition cold‐dry air? Two separate studies, de Azevedo et al. (2017) and Wroe et al. (2018), have used Computational Fluid Dynamics (CFD) analyses to evaluate Neandertal nasal air‐conditioning capabilities. Both studies concluded that Neandertal noses would have indeed been able to effectively heat and humidify cold‐dry air, showing that Neandertals possessed air‐conditioning capacities similar to those of European modern humans. Further, Wroe et al. (2018) specifically point out that Neandertals would have been able to achieve these comparable levels of air‐conditioning despite being able to transmit nearly twice as much air in each breath as a living modern human. Here, it should be noted that, because the fragile nasal turbinates (conchae) are damaged in virtually all Neandertal fossils, both of these CFD studies relied on models in which Neandertal turbinates were reconstructed based on those of European‐derived modern humans. The lack of preserved bony turbinates has long impeded assessments of Neandertal nasal function, as the turbinates greatly impact the SA:V ratio of the internal nasal cavity, and thus, its capacity for heat and moisture exchange. Indeed, Marks et al. (2019) have shown that modern humans from the Arctic Circle typically possess larger turbinates than populations from equatorial regions, a pattern consistent with climatically adaptive expectations based on SA:V ratios. Excitingly, recent endoscopic investigations (Buzi 2020; Buzi et al. 2024) of the perfectly preserved nasal cavity of the Altamura 1 Neandertal cranium (which is irretrievably embedded by flowstone in Lamalunga Cave near Altamura, Italy), have provided the first glimpses of intact Neandertal turbinates—and following cold‐adapted expectations—they appear to be incredibly large. Indeed, as initially predicted by Steegmann et al. (2002), the turbinates of Altamura 1 appear to “crowd” the inside of the nasal cavity, suggesting they would have greatly enhanced the specimen's ability to heat and humidify air. Accordingly, future CFD studies incorporating morphological data from the Altamura 1 specimen may eventually reveal that Neandertals were even better adapted for breathing cold‐dry air than currently recognized.

## 5 Neandertal Biological Adaptations to Cold: Physiological Cold Adaptation

Physiological cold climate adaptations are often difficult to infer from the fossil record, as direct evidence of thermoregulatory processes is scarce. Consequently, our theoretical understanding of Neandertal physiological adaptations to cold is largely derived from studies of modern humans inhabiting similar climates. Any such application of modern human physiological studies to Neandertals must, of course, come with the caveat that no cold climate population should be considered fully representative of Neandertals in terms of physiology or lifeways. Rather, studies of modern humans in cold climates merely provide us with a non‐comprehensive suite of physiological possibilities that may have been available to Neandertals for surviving and thriving in their cold environments. Physiological assessments of cold climate adaptations have focused on vascular changes, basal metabolism, physical activity levels, diet, and brown adipose tissue (BAT) (non‐shivering thermogenesis). We will discuss each of these in turn.

The human body employs two primary vascular responses to cold: vasoconstriction and countercurrent heat exchange. To minimize the amount of heat lost to a cold environment, blood vessels in the extremities vasoconstrict, reducing blood flow to relatively high surface area limbs and effectively redirecting circulation to the body's warm core. This does, however, increase the risk of both inadequate nutrient delivery as well as cold‐related tissue damage in the extremities. To mitigate these risks, the body oscillates between vasoconstriction and vasodilation in a phenomenon referred to as the hunter's response, reducing the risk of both heat loss and damage to the extremities (Stocks et al. 2004; Steegmann Jr. 2007; Moran 2008). Countercurrent heat exchange similarly serves as an additional thermoregulatory mechanism by redirecting blood flow to the deep vessels of the extremities. In this way, warm blood from the core traveling through deep arteries transfers heat to the cooler blood returning through the deep veins. This limits the amount of heat lost from the body's core, enhancing overall heat retention (Huizenga et al. 2001).

Beyond minimizing heat loss, the body can also increase heat production. One way to accomplish this is to increase basal metabolic rate (BMR, kcal/day), the body's maintenance cost in the absence of other metabolic demands such as digestion or elevated immune function. Extensive research within human biology has been conducted to measure and estimate BMR across populations in a variety of climates. While BMR is most strongly correlated with fat‐free mass, substantial interpopulational and interindividual variation has been documented (Johnstone et al. 2005; Manini 2010; Dugas et al. 2011). Despite this observed variation, we do see a noticeable pattern for modern circumpolar populations—a BMR ~8%–40% higher than predictive estimates—suggesting increased heat production in cold climates to maintain core body temperature. This elevated BMR is believed to be driven and maintained by higher thyroid hormone levels, with several studies providing supporting evidence (Danforth and Burger 1989; Tkachev et al. 1991; Levine et al. 1995; Ulijaszek 1996; Leonard et al. 2005). For example, it was found that Evenki, an indigenous group of reindeer herders living in Siberia, had significantly higher thyroid hormone levels and BMRs than predicted (Leonard et al. 1999, 2002; Galloway et al. 2000).

This work has inspired many researchers to apply what we have learned from modern cold climate populations to Neandertals. One of the earliest attempts to estimate Neandertal BMR came from Sorensen and Leonard (2001), who estimated Neandertal BMR from estimated body mass, adding 10% in accordance with FAO/WHO/UNU guidelines for BMR estimates in cold climates (an increase in BMR by 3% for every 10°C below a reference temperature of 10°C; FAO/WHO/UNU 1985). Their calculations yielded an estimate of 1435 kcal/day for female Neandertal BMR and 1841 kcal/day for males. Building on this, Froehle (2008) incorporated estimates for body mass (using Ruff et al. 1997), fat free mass, and relevant environmental variables to develop more environmentally specific predictive equations for Neandertal BMR. Froehle's estimates for Neandertal BMR range from 1446 to 1450 kcal/day for females and 1788–1951 kcal/day for males, while anatomically modern human BMR was estimated to be 1351–1509 kcal/day for females and 1628–1902 kcal/day for males. Seasonal estimates have also been calculated, suggesting that in summer and then in winter, Neandertal females would have had a BMR of 1465 and 1758 kcal/day, respectively. For males, they estimated a summer BMR of 1876 and 2251 kcal/day for winter (Snodgrass and Leonard 2009).

As an interesting aside, among modern humans, there appears to be a positive correlation between BMR and blood pressure (Luke et al. 2004) as well as a marked increase in blood pressure during cold exposure due to a combination of increased metabolism and increased vasoconstriction (Kingma et al. 2011). This raises the intriguing possibility that Neandertals, given their likely high BMRs and cold climate inhabitance, may have had elevated blood pressures. Alternatively, and perhaps even more intriguing, they may have had a unique physiological adaptation that avoided high blood pressure and its associated complications, such as retinal damage, cardiovascular disease, and kidney damage. Investigating potential genetic signatures of such an adaptation could be a compelling avenue for future research.

Another key mechanism for heat production is physical activity due to the inefficiency of human muscle. Neandertal locomotion and physical activity levels have been a topic of much discussion within biological anthropology. The aforementioned shortened lower limbs of Neandertals combined with the rugged terrain they inhabited and navigated suggest that they had high levels of physical exertion. Research among modern humans has demonstrated that locomotor cost is inversely related to lower limb length (Steudel‐Numbers and Tilkens 2004; Pontzer 2007a, 2007b). Based on this principle, Steudel‐Numbers and Tilkens (2004) estimated that Neandertals would have expended approximately 30% more than their longer‐limbed anatomically modern human counterparts. This has been interpreted as an energetic disadvantage, perhaps opening the door to be outcompeted by anatomically modern humans. However, there are two key caveats to this thinking. First, while not yet empirically tested, it has been suggested that the shorter Neandertal distal leg elements would have improved locomotor efficiency on rugged, uneven terrain—the very kind the Neandertals traversed (Higgins and Ruff 2011). Second, muscle contraction is inherently inefficient: roughly 80% of the energy used during muscle contraction is lost to heat while 20% is converted to work (Hargreaves and Spriet 2020). As such, muscle contraction through physical activity can contribute meaningfully to thermoregulation in cold conditions. If Neandertals did indeed have high physical activity levels in conjunction with relatively high muscle mass, this muscular inefficiency could have been a thermoregulatory boon in glacial climates, reframing the way we have traditionally thought about Neandertal supposed inefficiency.

Neandertals have often been characterized as predominantly meat eaters (Richards et al. 2000, 2001, 2008; Richards and Schmitz 2008; Bocherens 2009; Gaudzinski‐Windheuser and Kindler 2012). However, more recent work has demonstrated that, like all humans, Neandertal diets varied seasonally and geographically (Aranguren et al. 2007; Richards and Trinkaus 2009; Hardy and Moncel 2011; Dodat et al. 2024). Nonetheless, Neandertals likely consumed a greater proportion of calories from mammalian and marine sources given the reduced bioavailability of plant sources in higher latitudes compared to lower ones (Garibotto et al. 2010). Diets that regularly exceed ~35% protein can lead to protein toxicity and potential kidney damage (Cordain et al. 2000; Hardy 2010; Fiorenza et al. 2015). Available data suggest Neandertals consumed more protein on average than is recommended for humans today, presenting the possibility that Neandertals possessed a unique physiological mechanism for avoiding protein toxicity.

This discussion is physiologically important because a high protein diet can contribute to thermoregulation through the thermic effect of food (TEF), which is the metabolic cost of digestion. TEF typically accounts for 10% of total caloric intake, and it is determined not only by the number of calories consumed in a meal, but also by the macronutrient content of that meal (Kinabo and Durnin 1990; Reed and Hill 1996). Protein increases the metabolic rate associated with TEF by approximately 30%, whereas carbohydrates and fats increase it only by ~5%. Furthermore, the metabolic elevation associated with protein intake can persist up to 12 h after the meal has been consumed, whereas the metabolic bump from carbohydrates and fats only lasts a few hours (Reed and Hill 1996; Farshchi et al. 2004; Halton and Hu 2004). This increased metabolism associated with high protein intake would have been beneficial for Neandertals. Though the elevated protein TEF could be viewed as inefficient, since fewer of the calories are going to body maintenance, growth, and reproduction, the increased TEF also produces more heat that would effectively lower thermoregulatory costs in a cold environment. The impact of a high protein diet on Neandertal metabolism, potentially kidney health, and thermoregulation has yet to be fully explored.

The final thermoregulatory feature, at least for this discussion, that may have been present among Neanderthals is BAT. BAT is a form of mitochondria‐dense adipose tissue that under acute cold stress produces heat rather than adenosine triphosphate (ATP) for work. This heat production is achieved through a short‐circuiting of the electron transport chain that results in the production and leak of protons (Cannon and Nedergaard 2012; Ouellet et al. 2012). Among adult humans, BAT is found in the supraclavicular region, potentially to provide thermoregulatory protection to the carotid arteries and jugular veins, as well as along the major deep blood vessels such as the thoracic and abdominal aorta (Cannon and Nedergaard 2012; Ouellet et al. 2012). Though originally thought to only exist in human babies and hibernating mammals, recent studies have demonstrated BAT activity among adult humans in cold climates like Siberia (Levy et al. 2018) and northern Finland (Ocobock et al. 2022), temperate climates (van der Lans et al. 2016; Cannon and Nedergaard 2012; Ouellet et al. 2012; Levy 2019; Niclou and Ocobock 2020), and even in tropical Samoa (Niclou et al. 2024). This broad distribution suggests that BAT is pervasive throughout humans and may well be evolutionarily ancient—likely present in Neandertals.

Among Finnish reindeer herders exposed to acute cold (~12°C–15°C, for 30 min), BAT increases the metabolic rate an average of 8.7%. Additionally, the BAT positive region (supraclavicular) experienced only a 4.6% decline in surface temperatures, whereas the BAT negative regions (sternum) exhibited a mean temperature decline of 10.5% (Ocobock et al. 2022). Given its thermogenic properties, BAT could have been critical to Neandertal survival in glacial regions, helping to maintain core body temperature by insulating the life‐sustaining major blood vessels. However, recent genetic research has not supported this hypothesis. For example, a recent study looked at a variant of the rs1421085 T>C gene, where the C allele enhances BAT activity by increasing the expression of UCP1—the enzyme responsible for uncoupling the electron transport chain and resulting in the production of heat rather than ATP. The study also looked at the presence of a homologous variant across human populations and found an inverse correlation between the BAT‐enhancing variant and ambient temperature. The investigators also suggested this allele came about roughly 780–130 ka, well within the window of Neandertal appearance. This variant has not been detected in the Neandertal genome; however, that does not mean there isn't a similar variant homologue within Neandertals that has not yet been identified (Yin et al. 2024).

A common theme throughout this suite of potential Neandertal physiological cold adaptations is an exceptionally high metabolism. In order to fuel their higher BMR, physical activity levels, protein‐heavy diet, and thermoregulatory mechanisms like BAT, Neandertals would have had incredibly high total energy expenditures (TEE, kcal/day). Much like BMR, this too has been estimated, placing female Neandertal TEE within ~3200–5200 kcal/day and male Neandertal TEE within ~4500–6750 kcal/day (Churchill 2006; Froehle and Churchill 2009; Snodgrass and Leonard 2009). For comparison, Hadza hunter‐gatherer females from northern Tanzania typically range from 1459 to 2956 kcal/day and males 2008–3363 kcal/day while Bolivian horticulturalists ranged from 1972 to 3202 kcal/day and 2212–3374 kcal/day for females and males, respectively (Pontzer et al. 2012). So, even the low end of the Neandertal TEE estimates put them well above the high end for modern‐day hunter‐gatherers and horticulturalists. This kind of high metabolic throughput could only have been sustained through highly successful acquisition of resources in combination with an anatomy (the large chest and nose that enable high oxygen flow) and physiology uniquely adapted to the rigorous demands of glacial environments.

## 6 Summary and Conclusions

In Pleistocene western Eurasia, Neandertals repeatedly faced cold stress and responded with a mosaic of cultural buffers, developmental responses, and inherited biology. Considering evidence across technology, acclimatization, development, and genetics helps address the central question of cold adaptation without reducing it to any single trait.

Cultural adaptations characteristic of the human niche encouraged technological strategies that likely shielded Neandertals from their challenging environments. Hearths were probably common, and claims of birch‐tar production may point to sophisticated fire management. Toolkits associated with hide working (e.g., scrapers, lissoirs), along with the presence of eyed needles at Denisova, suggest clothing that, while likely loose in many regions, may have also included tailored garments in colder settings. Collectively, these behaviors and advances would have mitigated exposure and reduced energetic costs.

Anatomically, Neandertals exhibit the classic cold‐adapted pattern: high body mass, shortened limbs, and broad pelves. Yet development and population history may complicate adaptationist interpretations. Distal limb shortening can arise from repeated cold‐induced vasoconstriction during growth, and Neandertal fetuses already show this foreshortening. Selection likely acted on phenotypes partly shaped by plasticity, with trunk breadth and thoracic integration playing a greater thermoregulatory role than distal limb proportions alone.

The Neandertal nose has been described as a paradox with a strongly projecting external nose combined with a wide aperture. However, a respiratory–energetics framework predicts large nasal dimensions in large‐bodied, high‐throughput foragers; CFD modeling shows Neandertal nasal cavities could heat and humidify cold‐dry air comparably to European *H. sapiens* while transmitting substantially greater airflow. Though the internal nose is rarely preserved in Neandertals, the first endoscopic views of Altamura 1 suggest they possessed strikingly large turbinates, consistent with enhanced internal air‐conditioning.

Direct evidence of physiology is limited, but modern human studies provide plausible analogs. BAT–mediated thermogenesis is widespread among contemporary humans and measurably elevates metabolism in cold conditions. Estimated Neandertal total energy expenditures far exceed those of recent foragers, aligning with the anatomical picture of high metabolic demands.

In sum, Neandertals were cold adapted not through a single hallmark, but through an integrated suite of traits: cultural buffering (fire, garments), body geometry that reduces relative surface area, internally complex and capacious nasal airways for conditioning high‐flow, cold‐dry air, and likely physiological mechanisms to conserve and generate heat. Each of these domains was modulated by geography, development, and phylogenetic constraint. This integrated perspective helps explain Neandertals' ecological breadth and their persistence across varied climates for over one hundred thousand years.

## 7 Future Directions

Neandertals show a host of features likely indicative of cold adaptation, from large body mass, wide pelves, voluminous ribcages, and abbreviated limb proportions, to the complex architecture of their internal noses. Given their close phylogenetic relationship with *H. sapiens* , we can assume that they share many physiological thermoregulatory adaptations with us, or perhaps have evolved their own analogous versions of these adaptations. As we learn more about the specifics of the Neandertal genome, we may be able to identify such adaptations. This is truly an exciting time to be researching Neandertals.

## Ethics Statement

The authors have nothing to report.

## Data Availability

Data sharing not applicable to this article as no datasets were generated or analysed during the current study.
